# Extra Skeletal Ewing's Sarcoma: a Case Report

**DOI:** 10.30476/DENTJODS.2019.44908

**Published:** 2020-03

**Authors:** Hassan Mir Mohmmad Sadeghi, Fatemeh Mashhadi Abbas, Nasim Taghavi, Nazan Baharnoori

**Affiliations:** 1 Dept. of Oral and Maxillofacial Surgery, Dental School, Shahid Beheshti University of Medical Sciences, Tehran, Iran; 2 Dept. of Oral and Maxillofacial Pathology, School of Dentistry, Shahid Beheshti University of Medical Sciences, Tehran, Iran; 3 Postgraduate Student, Dept. of Oral and Maxillofacial Pathology, Dental School Shahid Beheshti University of Medical Sciences, Tehran, Iran

**Keywords:** Ewing's Sarcoma, Extra Skeletal, Oral cavity

## Abstract

Ewing's sarcoma is an uncommon malignancy primarily affecting bone tissue and usually occurs in adolescents and young adults. This paper reports a rare case of extra-skeletal Ewing’s sarcoma of the oral cavity soft tissue. In the clinical examination, a mass of 1.5×1.5cm in diameter was observed in the right mandibular vestibule. Radiographic examination revealed no involvement of mandible. Microscopically, the tumor was composed of malignant small round cell tumor that exhibited immunoreactivity for CD99. Enucleation surgery under local anesthesia was performed for the patient and prognosis was excellent. The patient remained symptom-free after 13 months of follow-up.

## Introduction

Ewing’s sarcoma (ES) which is first described by James Ewing in 1921 is a malignancy primarily affecting skeletal system and it is commonly occurring in adolescents and young adults [ 1 - 2 ]. Overall, ES has an aggressive behavior with rapid growth and high probability of metastasis at time of diagnosis [ 3 ]. ES arising from soft tissue is referred to as an extra-skeletal Ewing’s sarcoma (EES). Primary EES in head and neck is uncommon and only five cases from 118 cases of EES are reported in head and neck region [ 4 ].

Based on the results of previous studies, the incidence of ES in the head and neck region is uncommon, when it occurs; it generally involves the mandible and less frequently the maxilla [ 5 - 6 ]. The present study describes a case of EES of soft tissue overlying mandibular body. 

## Case Report

A 27-year-old female with 10-month history of a gradually increasing intraoral swelling visited the oral and maxillofacial surgery ward of private hospital, Tehran, Iran. According to the patient's remark, the swelling has become firm in the last two months. There was no relevant medical or family history or a history of trauma, smoking, alcohol, addiction, and medication usage. On extraoral examination, there was no swelling and asymmetry. In addition, there was no lymphadenopathy.

**Figure1:**
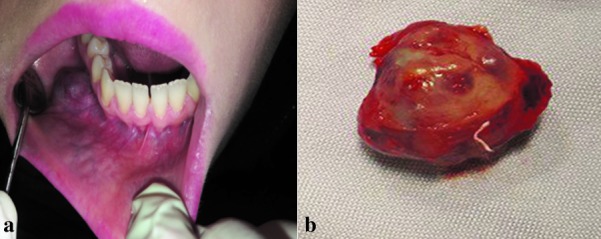
**a:**Intraoral examination revealed exophytic dome shape mass, **b:** Gross appearance

Intraorally, a removable pinkish-red dome shape exophytic lesion measuring 1.5×1.5cm in the right mandibular vestibule, in the region of right first and second premolar was noticed (Figure 1a). 

On palpation, the swelling was non-tender, rubbery in consistency with telangiectatic vessel on its surface and no ulceration was noted. Clinically the lesion resembled to benign salivary gland tumor. Panoramic radiograph showed no involvement of mandible. Aspiration was performed and the result was negative. The mass was enucleated under local anesthesia via vestibular incision with maintenance branches of mental nerve. Tissue was sent for histopathological examination.

### Gross characteristics and histopathology findings

**Figure2:**
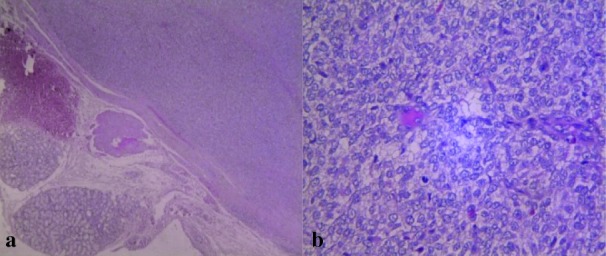
Histological sections show **a:** encapsulated lesion, H&E stain 40X **b:** small round cells with polymorphism, hyperchromatic nuclei; H&E stain 400X

Grossly, the tumor was well-circumscribed and encapsulated (Figure 1b). The H & E sections showed encapsulated malignant round cell tumor in which the cells revealed polymorphism, hyperchromatic nuclei, conspicuous nucleoli, and mitosis. Around the fibrous capsule, sections of minor salivary gland were also seen. The light microscopical diagnosis was a malignant small round cell tumor (Figure 2). Consequently, differential diagnosis including malignant lymphoma, malignant salivary gland tumor, and ES were considered.

### Immunohistochemistry

Considering that immunohistochemistry as the mainstay of diagnosis, and in order to rule out other malignancies, immunohistochemistry assessments were performed. 

**Figure3:**
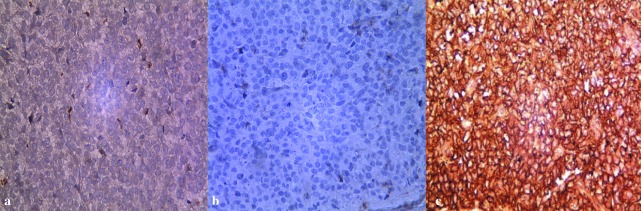
Immunohistochemical expression showing, **a:** negative for P63 (400X), **b:** negative for LCA (400X), **c:** CD-99positivity in cytoplasm of tumor cells (400X)

The tumor cells showed membrane positivity for CD 99 and were negative for leukocyte common antigen (LCA) and P63. These features were conclusive of ES (Figure 3).

### Magnetic resonance imaging (MRI) and whole body scan findings

**Figure4:**
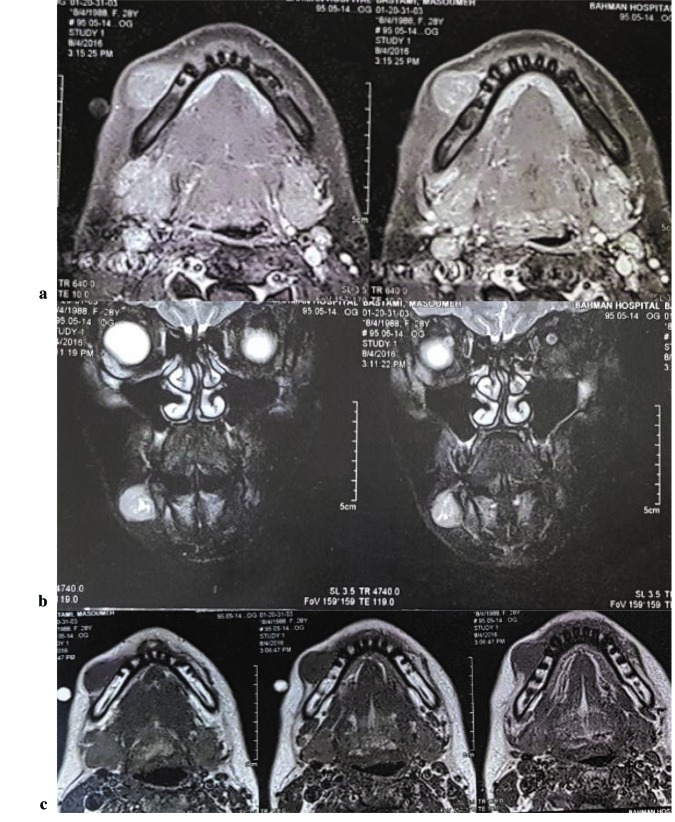
MRI scans show a well-defined soft tissue mass in the right side of mandible. **a:** T1 without Gadolinium, **b:** T1 with Gadolinium, **c:** T2 with Gadolinium

**Figure5:**
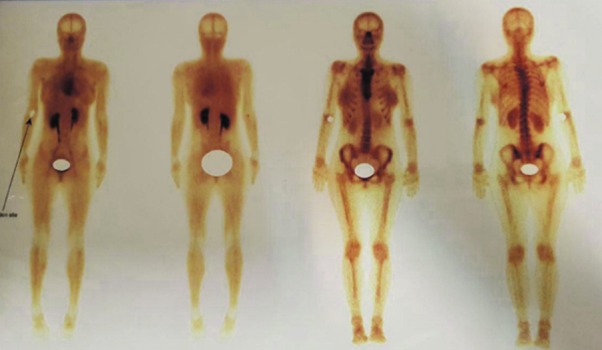
Whole body scans after IV administration of (740 MBq) 99mTc-MDP, revealed no metabolically active bone lesion

In order to rule out metastatic origin of tumor, whole body scan and MRI with/ without gadolinium were carried out. A well-defined mass in subcutaneous region over the right mandibular bone were observed. The tumor was high signal and heterogeneous on T2W images. On T1W images, the tumor was isointense to the muscles and on T1W post-contrast scans, heterogeneous enhancement was seen (Figure 4). In addition, whole body scan was made and no abnormalities were found (Figure 5). The follow-up of this case for past 9 months revealed no recurrence until now.

## Discussion

EES is a rare, rapidly growing malignant neoplasm that predominantly affects adolescents and young adults between 10 and 30 years of age, whereas the disease is distinctly uncommon in individuals before age 5 and after age 30 [ 7 - 8 ]. Overall, soft tissues of the extremities (particularly lower extremities), trunk, paravertebral, intercostal area, head and neck regions, pelvis, and retroperitoneum are more commonly involved in EES. ES arising from the soft tissue of oral cavity is exceedingly unusual [ 9 - 10 ]. In this report, we present a case of ES of the soft tissue of oral cavity and we believe this is the second case of EES of soft tissue of oral cavity.

High-resolution computed tomography (CT) and MRI scans are not specific but useful in the diagnosis and treatment planning. In the present study, we preformed MRI with/without gadolinium and whole body scan in order to rule out metastasis. The MRI features revealed isointensity to the muscles on T1W and heterogeneous enhancement on T1W postcontrast scan. These findings were similar to previous studies [ 11 ].

Biopsy is necessary to make a definitive diagnosis, and the tumor must be distinguished from similar tumors and conditions [ 12 ]. For this purpose, in this case immunohistochemistry has been performed and the results were negative for LCA (lymphomas) and P63 (Myoepithelial carcinoma) and it was positive for CD99, therefore, the diagnosis were confirmed. On the other hand, immunohistochemical assessments in some studies revealed positivity for pancytokeratin [ 13 ]. In addition, some molecular pathology techniques showed that transcription-polymerase chain reaction (RT-PCR) and fluorescence in situ hybridization (FISH) are valuable for evaluation of undifferentiated small round-cell tumors like ES [ 14 - 15 ].

Depending on the site of the tumor and its extension, treatment of EES includes multimodality approach consisting surgery, chemotherapy and radiotherapy. Overall, the prognosis of ES is poor [ 7 ]; our patient prognosis was excellent and responded favorably to enucleation surgery and after 13 months remained symptom free.

Informed consent was obtained for publishing her clinical photography and radiography.

## Conclusion

Although EES is quite rare, it should be take into consideration as differential diagnosis of intraoral masses. The present study highlights a rare case of EES in third decade of life and presented as mass in soft tissue of right mandibular vestibule with excellent prognosis and without recurrence.
